# The Neuropeptide Pituitary Adenylate Cyclase-Activating Polypeptide (PACAP) Is Protective in Inflammation and Oxidative Stress-Induced Damage in the Kidney

**DOI:** 10.3390/ijms20194944

**Published:** 2019-10-07

**Authors:** Gabriella Horvath, Balazs Opper, Dora Reglodi

**Affiliations:** Department of Anatomy, MTA-PTE PACAP Research Team, University of Pecs Medical School, 7624 Pecs, Hungary; gabriella.horvath@aok.pte.hu (G.H.); balazs.opper@aok.pte.hu (B.O.)

**Keywords:** PACAP, drug-induced nephropathy, renal ischemia-reperfusion, cytoprotection, cytokine expression

## Abstract

Pituitary adenylate cyclase-activating polypeptide (PACAP) is a pleiotropic neuropeptide with a widespread distribution throughout the entire body including the urinary system. PACAP exerts protective actions in different injury models related to several organ systems. Its protective effect is mainly based on its antiapoptotic, anti-inflammatory and antioxidant effects. The present review aims to summarize the effects of PACAP in pathologies associated with inflammation and oxidative stress-induced damage in the kidney. Both in vitro and in vivo data are available proving its protective actions against oxidative stress, hypoxia, renal ischemia/reperfusion, diabetic nephropathy, myeloma kidney injury, amyloidosis and different types of drug-induced nephropathies. Data showing the nephroprotection by PACAP emphasize the potential of PACAP’s therapeutic use in various renal pathologies.

## 1. Introduction

Pituitary adenylate cyclase-activating polypeptide (PACAP) was first isolated as a hypothalamic peptide based on its efficacy to activate adenylate cyclase in rat hypophyseal cells [1]. It is a member of the secretin/glucagon/vasoactive intestinal polypeptide (VIP) family. PACAP exists in two forms: PACAP38 and PACAP27, with 38 and 27 amino acid residues, respectively [2]. It acts through three receptors belonging to G protein-coupled receptor group. The specific PAC1 receptor binds PACAP with much higher affinity than VIP, while VPAC1 and 2 receptors show similar affinity to PACAP and VIP. It has a widespread distribution throughout the entire body both in the central nervous system and peripheral organs [2]. Both the peptide and its receptors are present in the urinary system [3,4]. PACAP plays various roles in the urinary tract. Among others, it stimulates renin secretion [5], influences renal blood flow [6,7] and affects the urinary bladder epithelium [8] and afferent pathways from the bladder [9].

Most data support the peptide’s protective effects in the kidney. PACAP was first identified as a neuroprotective peptide, with subsequent studies supporting its general cytoprotective actions [10,11,12,13,14]. The neuroprotective effects were first proven in neuronal cell cultures exposed to various toxic agents or growth factor withdrawal [2,15,16,17,18]. Later, in vivo data came from models of cerebral ischemia in rats and mice, where PACAP could significantly reduce the damaged brain area [19,20]. Similar neuroprotective effects were shown in neuronal trauma, spinal cord injury, models of Parkinson’s disease and Alzheimer’s disease as well as autoimmune encephalopathy and different retinal pathologies [21,22,23,24,25]. In the nervous system, several protective mechanisms have been identified, with antiapoptotic, anti-inflammatory and antioxidant effects accounting for the majority of the observed positive effects. The neuroprotective actions of PACAP have been reviewed extensively in several review papers [26,27,28,29,30]. Accumulating evidence shows that PACAP is not only protective in neurons but exerts general cytoprotective actions in various cell lines in vitro and in numerous injury models in vivo. Data indicate that not only exogenously administered PACAP, but also the endogenously present peptide is able to exert these effects. Among others, PACAP has protective effects in liver [31] and intestinal ischemia [32], inflammatory lung and skin conditions [33,34], has cardioprotective effects in various models of cardiomyocyte damage [35,36] and protects pancreatic beta cells [37]. Of the peripheral organs, most data have been accumulated on the protective effects of PACAP in the kidney against various lesions, both in vitro and in vivo [3,38]. The aim of the present study is to provide an updated review on the nephroprotective effects of PACAP. Based on the data summarized here, PACAP has a potential therapeutic use in various renal pathologies.

**Figure 1:**
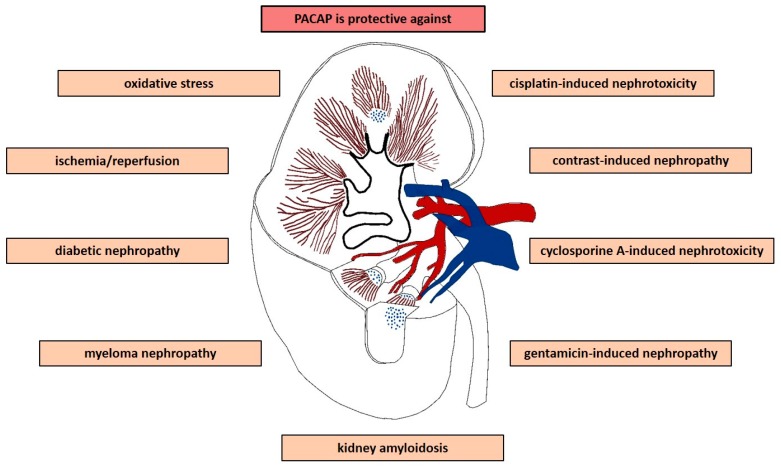
Summary of renoprotective effects of PACAP. List of renal pathologic conditions, in which protective effect of PACAP was evaluated.

Renoprotective effects of PACAP are summarized in Figure 1 and Table 1.

## 2. Effects of PACAP In Vitro

### 2.1. Oxidative Stress and Hypoxia

Oxidative stress plays a substantial role in many different renal pathologies. Reactive oxygen species are involved in the pathogenesis of various kidney diseases, such as diabetic nephropathy, aging, hypertension, ischemia/reperfusion injury or drug-induced nephropathy [57,58]. Previous studies revealed renoprotective effect of both exogenous and endogenous PACAP. The effect of PACAP on cell viability was examined in primary renal cell culture obtained from newborn rats [39]. Oxidative stress was induced by hydrogen peroxide solution leading to decreased cell survival. This change in cell viability could be counteracted by simultaneous treatment with 10 or 100 nM PACAP. Investigating the lowest effective dose of PACAP, it was found that PACAP exerted protective actions already at 100 pM concentration. In addition, Li et al. [44] investigated the effect of PACAP against in vitro hypoxia in proximal tubule epithelial cells isolated from wild type and MyD99-/- mice. In vitro hypoxia evoked by immersing the epithelial monolayer in mineral oil led to cytokine activation and cell death. Significantly increased levels of monocyte chemoattractant protein-1 (MCP-1), interleukin-6 (IL-6) and macrophage inflammatory protein-2 (MIP-2) were detected. These hypoxia-induced changes were suppressed by PACAP treatment.

Functions of endogenous PACAP can be examined in PACAP-deficient mice. Animals lacking endogenous PACAP have been revealed to display several abnormalities in development, behavior, inflammatory reactions and increased vulnerability against various stressors [59,60,61]. Early studies revealed that cerebellar granule cells isolated from PACAP-deficient mice were more sensitive to oxidative stress than cells from wild type animals [62]. This increased sensitivity has since been shown in many different in vitro and in vivo models of cellular and tissue injuries [63]. In agreement with this general endogenous effect of PACAP, increased vulnerability of renal cells lacking endogenous PACAP has been shown against hydrogen peroxide induced oxidative stress [64]. Cells were isolated from PACAP-/- mice and their data were compared to that of primary renal cell culture of wild type animals. The injury caused by in vitro oxidative stress could be attenuated by administration of exogenous PACAP. These results were in accordance with other observations exploring the sequelae of the lack of PACAP in various organs and cells in different models [63]. These studies were completed by in vitro hypoxia [65]. Cells derived from wild type and PACAP-/- mice were exposed to CoCl_2_-induced in vitro hypoxia. Similarly to previous observations regarding the experiments of oxidative stress, mice lacking PACAP responded to in vitro hypoxia with higher vulnerability.

### 2.2. Effect of PACAP against Drug-Induced Nephropathy

#### 2.2.1. Effects of Exogenous PACAP against Gentamicin-Induced Nephrotoxicity In Vitro

Aminoglycoside antibiotics are common causes of drug-induced nephropathy. The excreted antibiotic accumulating in proximal tubule leads to nephrotoxicity. Gentamicin in 2 µg/mL concentration leads to significant decrease in cell survival [41]. PACAP treatment was shown to alleviate the survival-worsening effect of gentamicin. In order to get insight in the mechanism of PACAP, a kidney biomarker assay was performed. PACAP could counteract the expression-decreasing effect of gentamicin on dipeptidyl peptidase IV and vascular endothelial growth factor.

#### 2.2.2. Cisplatin Nephrotoxicity

Nephrotoxicity is a common side effect in patients receiving cisplatin chemotherapy. Twenty percent of the patients show severe renal dysfunction and approximately one-third of the patients treated with high dose cisplatin suffer from signs of kidney injury [66]. The main pathological elements in cisplatin nephrotoxicity are renal tubular cell injury and death. A robust inflammatory reaction and activation of inflammasomes are also stimulated, further exacerbating renal damage [67]. In vitro studies confirmed the renoprotective effect of PACAP against cisplatin-induced damage [68]. Human proximal tubule epithelial cells (HK-2) were exposed to 50 µM cisplatin leading to increased tumor necrosis factor alpha (TNF-alpha) secretion, which could be alleviated by PACAP treatment in a dose-dependent manner. The background mechanism of cisplatin-induced changes involve activation of p53, caspase-6, caspase-7, poly (ADP ribose) polymerase-1 (PARP-1) and suppression of apurinic/apyrimidinic endonuclease-1 (APE-1). The treatment with PACAP significantly reduced the p53-dependent transcriptional control of caspase-7 and PARP-1 in nuclear extracts and partially restored the APE-1. Furthermore, western blot examinations revealed that PACAP was able to increase Bcl-2 (B-cell lymphoma-2) and Bcl-XL (B-cell lymphoma-extra large) and to decrease Bax (Bcl-2 associated X protein) after their cisplatin-induced changes [68]. In addition, authors have identified mRNAs of SV1, SV2 and short variants of PAC1 receptor and VPAC1 receptor both in control and cisplatin-treated HK-2 cells. Cisplatin exposure led to significant reduction of PAC1 expression and it almost abolished the expression of VPAC1-R, while treatment with PACAP38 increased the PAC1 receptor expression. If cells were treated with both cisplatin and PACAP38, no change in receptor expression was detected [68]. Li and colleagues [42] extended their experiments on PACAP’s renoprotective effect using primary mouse renal proximal tubular epithelial cell culture. Supporting their previous results of experiments performed in HK-2 cells, similar effect of PACAP could be detected assessed by annexin V/propidium iodide staining. PACAP treatment led to significant increase in number of viable cells exposed to 50 µM cisplatin. Besides exploring effective agents for reducing severe side effects of chemotherapeutic, another direction was studied. Garofalo et al. [69] developed experiments investigating the biodistribution of extracellular vesicles, which could be possible delivery systems for chemotherapeutic agent with lower incidence of systemic side effects.

#### 2.2.3. Cyclosporine A-Induced Nephrotoxicity

Cyclosporine A is a potent immunosuppressant used for preventing allograft rejection in solid organ transplantation. Since its introduction, it improved substantially the overall success rates after solid organ transplantation [70]. Despite the beneficial effect on allograft rejection and in autoimmune diseases, cyclosporine A treatment can lead to impaired renal function [71]. Khan et al. explored PACAP’s effect in cyclosporine A-induced nephrotoxicity in proximal tubule epithelial cells [40]. HK-2 cells were treated with 50 µM cyclosporine A in the presence or absence of PACAP. Cyclosporine A-treatment led to elevation of transforming growth factor beta-1 (TGF-beta-1) level, which could be attenuated by PACAP pretreatment. Cell morphology was also assessed by phase-contrast microscopy. HK-2 cells receiving cyclosporine treatment showed discernible morphological changes. After 24 h, cells began to show elongated fibroblast-like morphology. These morphological changes could be prevented by PACAP pretreatment, but not by simultaneous PACAP addition. These results were supported by lactate dehydrogenase (LDH) cytotoxicity assay. Nephrotoxic effect of cyclosporine A led to significant release of LDH into the culture medium. This increased LDH release was reduced to baseline with PACAP treatment if PACAP was added 1 h prior to cyclosporine A treatment. In addition, PACAP exerted apoptosis-inhibiting effect in cyclosporine A-treated HK-2 cells as assessed by determination of cytoplasmic histone-associated DNA fragmentation. PACAP was also able to reduce the intracellular concentration of reactive oxygen species in cyclosporine A-treated HK-2 cells.

#### 2.2.4. Contrast-Induced Nephrotoxicity

Contrast-induced nephropathy is relatively common in patients suffering from preexisting kidney or circulatory disease e.g., diabetic nephropathy, congestive heart failure and impaired renal function [72]. Contrast media molecules are freely filtered through the glomerular filter layers and without reabsorption they present large osmotic pressure in the renal tubules. Impaired renal function aggravates the elimination of contrast media causing structural changes in renal tubules with further deterioration of renal function [72]. Khan and colleagues investigated the effect of PACAP in contrast-induced nephropathy [73]. Both ionic (Urografin) and nonionic (iohexol) contrast media were tested. Urografin led to significant apoptosis in HK-2 human proximal tubule epithelial cells. This decreased cell viability could be counteracted by PACAP pretreatment in a dose-dependent manner. PACAP was also able to offset the proliferation-inhibiting effect of Urografin and iohexol. In addition, PACAP reduced the contrast-medium-evoked elevated release of kidney injury molecule-1 (KIM-1) into the culture medium.

### 2.3. Myeloma Kidney Injury

Arimura et al. [45] investigated the effectiveness of PACAP against myeloma kidney injury in vitro. Cultured proximal tubular epithelial cells were exposed to kappa light chains isolated from the urine of a patient suffering from multiple myeloma. PACAP38 could decrease the kappa light chain-induced proximal tubule epithelial cell injury and elevated expression of IL-6 and TNF-alpha. PACAP receptor antagonists were used in order to clarify the receptor activation in the background of PACAP’s effect. Both M65, PAC1 receptor antagonist and PG97-269, VPAC1 receptor antagonist were able to attenuate the suppressive effect of PACAP on TNF-alpha production indicating that both receptor types are involved. PACAP was proven to act through influencing p38 mitogen-activated protein kinase (MAPK) activation.

### 2.4. Diabetic Nephropathy

Diabetic nephropathy is the leading cause of renal insufficiency [74]. Inflammation plays a relevant role in the pathogenesis and progression of diabetic nephropathy [75]. Sakamoto et al. [43] performed experiments aiming to model diabetic nephropathy in cultured mouse podocytes. Toll-like receptor 4 (TLR4) mediating the effects of lipopolysaccharide (LPS) was localized on podocytes and endothelial cells. Through TLR4, LPS led to significant increase of IL-6 and MCP-1. PACAP could attenuate the LPS-induced increased expression of the beforementioned proinflammatory cytokines, acting on VPAC-1 receptors through cyclic adenosine monophosphate (cAMP)/protein kinase A (PKA)-dependent signaling pathway. In addition, it significantly ameliorated the LPS-induced ERK phosphorylation and NF kappa B transnuclear localization. PCR examinations were performed in order to obtain further information on mechanism of PACAP’s effects. VPAC1, but not PAC1 receptor mRNA could be shown in cultured podocytes and isolated glomeruli [43].

## 3. In Vivo Protective Effects of PACAP

### 3.1. Ischemia/Reperfusion Kidney Injury

Ischemia/reperfusion kidney injury can be a primary or secondary cause of renal failure and is associated with inflammatory factors. There is accumulating evidence that PACAP is able to attenuate ischemia/reperfusion injury of the kidney. The protective effects of PACAP in ischemic lesions have been proven in the central nervous system and several peripheral organs. The first studies showing such potency of PACAP came from models of global and focal cerebral ischemia [19,76,77], where PACAP reduced size of the infarcted brain areas. Later this was confirmed in the retina, where bilateral carotid artery occlusion-induced retinal hypoperfusion was attenuated by PACAP administration [78]. Such efficacy of PACAP counteracting ischemic lesions has been subsequently proven in peripheral organs, including intestinal and renal ischemia/reperfusion injuries [32,79,80]. These findings have been summarized in review papers [3,38,80], so here we briefly address the protective effects of PACAP in ischemia/reperfusion kidney injuries, highlighting the effects on inflammatory and oxidative stress mediators, and describe more recent data.

The first proof of PACAP being nephroprotective in kidney ischemia came from results by Riera and coworkers [46]. They found that continuous infusion of PACAP resulted in nearly normal serum creatinine levels and reduced the morphological damage as shown by the tubulointerstitial injury. In addition, PACAP could elevate interleukin-6 levels and decreased myeloperoxidase activity as well as the CD45+ cell number, indicators of inflammatory cellular infiltration [46]. This study was followed by several others proving the protective efficacy of PACAP in in vivo ischemia/reperfusion injury. Szakaly et al. [47] demonstrated that a single PACAP injection markedly reduced the mortality following 45, 60 or 75 min ischemic periods. PACAP also ameliorated the morphological damage, as shown by the decreased level of tubular atrophy. PACAP could decrease the level of oxidative stress following 60 min ischemia, as demonstrated by the restored level of the antioxidant superoxide-dismutase [39]. Furthermore, PACAP injection led to an increased level of glutathione in spite of not affecting the level of malondialdehyde, a marker of oxidative stress. PACAP did not affect mitochondrial permeability but restored the reduced levels of the antiapoptotic Bcl-2 molecule. The markedly altered cytokine expression profile after ischemic injury was also partially reversed by PACAP treatment, such as fractalkine, intercellular adhesion molecule-1, RANTES, tissue inhibitor of metalloproteinase-1 and macrophage inflammatory protein [48]. Li and coworkers later showed that these effects are also present in a mouse model of ischemia/reperfusion [44]. Reduced tubular damage, apoptosis and cellular immune reaction were observed after PACAP treatment. TNF-alpha levels were also decreased, and kidney function was restored in the PACAP-treated groups. A subsequent study [81] investigated the involvement of toll-like receptors in PACAP-mediated nephroprotection in ischemia-reperfusion injury in a mouse model. Similarly to previous studies, PACAP-treated animals had maintained serum creatinine levels, showing normalized kidney functions. Kidney injury biomarkers were markedly reduced after PACAP treatment, as well as the ischemia-induced increase in TNF-alpha. Apoptosis and neutrophil accumulation were also reduced. Khan and colleagues showed dozens of toll-like receptor genes to be changed after ischemia, and found that PACAP treatment could reverse these changes, as well as suppress the toll-like receptor-associated cytokine protein levels [81].

In a more recent study, effects of PACAP were compared between female and male rats after ischemia/reperfusion. As most in vivo studies are performed in male rats, and PACAP exhibits several sex-dependent effects, it is of utmost importance to investigate whether the cytoprotective effects of the peptide can also be observed in females and whether there are any sex-dependent differences [49]. Male and female Wistar rats underwent the same procedure of unilateral renal artery clamping followed by 24-h, 48-h, or 14-day reperfusion. Rats received intravenous PACAP injection before the onset of ischemia. Tubular damage was markedly less severe in the PACAP-treated male and female rats compared to vehicle-treated controls, with female animals showing significantly better results in both treated and untreated groups. Cytokine expression, oxidative stress marker and antioxidative status confirmed the histological results. PACAP also counteracted the ischemia-induced decreased PKA phosphorylation, influenced the expression of BMP2 and BMP4, and increased the expression of the protein Smad1. These results could confirm that PACAP is protective in ischemia/reperfusion-induced kidney injury in both sexes, but females have less pronounced damage following ischemia/reperfusion, possibly also involving further protective factors.

Altogether, these studies clearly demonstrate that PACAP is able to exert protective effects in kidney ischemia/reperfusion injury; it reduces mortality, reduces structural damage and attenuates oxidative stress and inflammatory reactions in ischemic kidneys.

### 3.2. Diabetic Nephropathy

Diabetic nephropathy is one of the leading causes of end stage renal disease and is also one of the main complications of diabetes, along with diabetic retinopathy and neuropathy [82,83,84]. In a model of diabetic nephropathy diabetes was induced by a single intravenous injection of streptozotocin in male rats [52]. PACAP-treatment was given intraperitoneally every second day for 8 weeks. Detailed histological analysis of glomerular PAS positive area/glomerulus area, tubular damage and arteriolar hyalinosis revealed severe diabetic changes in kidneys of control diabetic animals (glomerular PAS-positive area expansion, tubular damage, Armanni-Ebstein phenomenon). PACAP treatment could markedly attenuate these morphological changes. Inflammatory reactions were evaluated by cytokine array. Diabetic kidneys showed a severe cytokine activation in comparison, while PACAP significantly downregulated numerous cytokines like CINC-1, TIMP-1, LIX, MIG, s-ICAM. This study showed that PACAP counteracts diabetes-induced changes in the kidney, at least partly through its well-known anti-inflammatory effect [52]. In another study, continuous infusion of PACAP for 2 weeks also ameliorated streptozotocin-induced diabetic changes in the kidney, including kidney weight, proteinuria and glomerular enlargement [53]. PACAP was also able to reduce the diabetes-induced increase in the proinflammatory TNF-alpha and the profibrotic TGF-beta.

In a follow-up study, molecular mechanisms responsible for PACAP-induced protective effects in a streptozotocin diabetic nephropathy model were further explored [54]. Diabetic animals showed an increase in the level of the proapoptotic pp38MAPK and cleaved caspase-3, effects counteracted by PACAP treatment. PACAP administration could also decrease the expression of p60 subunit of NF kappa B. The examined antiapoptotic factors, including pAkt and pERK1/2 showed a slight increase in the diabetic kidneys, while PACAP treatment resulted in a marked increase. Downregulation of fibrotic markers, like collagen IV and TGF-beta-1 in the kidney, was observed after PACAP treatment. Furthermore, PACAP treatment resulted in increased expression of the antioxidant glutathione, demonstrating the effect of PACAP in the system fighting oxidative stress. Segmental thickening of the glomerular basement membrane was also observed by electron microscopy in several parts of the diabetic glomeruli. PACAP-treated diabetic animals did not display this focal segmental thickening. In addition, severe podocyte injury was present in the diabetic glomeruli with marked foot process broadening and extensive flattening in contrast to the PACAP-treated kidneys. In summary, these studies show that PACAP has promising effects in diabetic nephropathy, acting through a complex mechanism including antiapoptotic, antifibrotic, antioxidative and anti-inflammatory actions [54].

### 3.3. Myeloma Kidney Injury

The above-described in vitro protective effects of PACAP against myeloma light chain protein could be confirmed in vivo [53,85,86]. Rats were treated with myeloma light chain and received intravenous PACAP injection, which abolished the increase in TNF-alpha. Interestingly, in spite of the general protective effects of PACAP, myeloma cell proliferation was suppressed both directly and indirectly through interleukin-6 suppression. Inhibition of growth in myeloma cells through IL-6 suppression is mediated through the human PAC1 receptor short subtype and/or VPAC2 receptor [85]. In a single patient case study, PACAP was confirmed to act against myeloma kidney injury [86]. A decrease in lambda light chains in the urine was measured through the 24-h observation period, in spite of unchanged other laboratory parameters.

### 3.4. Drug-Induced Nephropathy

#### 3.4.1. Gentamicin-Induced Nephropathy

In agreement with the positive in vitro findings, in vivo efficacy of PACAP against gentamicin-induced nephropathy has also been proven [53]. Renal toxicity caused by the accumulation of gentamicin in the proximal tubules was attenuated by repeated intravenous administration of PACAP in rats, as shown by the reduced production of the proinflammatory TNF-alpha [53].

#### 3.4.2. Cisplatin-Induced Nephropathy

Cisplatin, a widely used chemotherapeutic agent, has many side effects that can limit its clinical use. Among them, nephrotoxicity, neuro-and ototoxicity are the leading complications. PACAP has been proven to prevent cisplatin-induced neurotoxicity without affecting its chemotherapeutic action, as it did not affect the apoptotic pathways in proliferating ovarian cancer cells, but it protected neuronal cells [87]. Subsequent studies showed that PACAP is also able to reduce cisplatin-induced nephrotoxicity in a mouse model, in accordance with the above-described in vitro protective effects [68]. Mice were treated with PACAP 2 h before and 24, 48 or 72 h after cisplatin injection. Kidney functions were more preserved in PACAP-treated groups, shown by the reduced levels of serum creatinine and blood urea nitrogen [42]. The acute tubular damage was markedly less in the PACAP-treated animals: the corticomedullary tubular damage was reduced, less apoptotic cells were observed and PACAP could partially reverse the p53 response to cisplatin treatment. Furthermore, an amelioration could be observed in extracellular matrix components as well as PACAP could promote renal tubular cell regeneration [42]. These results provide evidence that PACAP could be a promising therapeutic agent in preventing cisplatin nephrotoxicity when given prior to treatments and can also attenuate kidney damage when administered after cisplatin injections [42].

#### 3.4.3. Contrast Agent-Induced Nephropathy

The protective effects of PACAP proven in an in vitro model system have also been demonstrated in vivo, in a mouse model [73]. Mice received intraperitoneal PACAP injection one hour before and 12 h after contrast agent administration, following water deprivation. The contrast agent Urografin induced severe tubular damage, apoptosis, increased oxidative stress and led to inflammatory reactions shown by the neutrophil granulocyte accumulation and proinflammatory cytokines. PACAP could effectively counteract all these changes: PACAP-treated animals had less tubular damage in the proximal tubules, fewer apoptotic cells and very few neutrophils in the cortex, altogether showing markedly reduced tubular damage. Lower levels of the inflammatory cytokines TNF-alpha, TGF-beta, interferon-gamma and macrophage chemoattractant protein-1 were observed after PACAP injection. A kidney biomarker assay further confirmed these actions, as several markers for kidney injury were reduced in PACAP-treated mice. PACAP treatment also increased antiapoptotic molecule expression, while proapoptotic markers were reduced. These changes led to an improved functional outcome proven by the restored serum creatinine levels. These results provide evidence that the peptide can be a potential nephroprotective agent in preventing contrast agent nephropathy, a common complication in the clinical practice [73].

#### 3.4.4. Cyclosporine A-Induced Nephrotoxicity

The clinical use of cyclosporine A, an immunomodulator, is often limited by its nephrotoxic effects. Similarly to the above-described protective effects of PACAP against numerous nephrotoxic agents, PACAP could restore kidney functions shown by the nearly normal serum creatinine levels. The severe tubulointerstitial damage was significantly reduced by PACAP treatment, evaluated by the loss of epithelial brush border, interstitial fibrosis, dilation of the tubules and intratubular debris and cast formation [40]. Changes in cell junctional markers, such as zonula occludens-1, were also restored by PACAP treatment as well as the increase in smooth muscle actin, indicative of myofibroblastic transformation. These results are also in accordance with PACAP restoring intercellular junctional properties of epithelial cells, as shown for example in retinal pigment epithelial cells against various stressors [88,89]. Collagen III and IV, as well as laminin and fibronectin extracellular matrix components were increased by cyclosporine A and decreased after PACAP treatment. Altogether, these results show that PACAP is able to counteract different damages induced by cyclosporine A treatment [40].

### 3.5. Kidney Amyloidosis

Given the antiapoptotic, anti-inflammatory and antioxidant effects of PACAP and its endogenous protective effects in several tissues, the question can be raised whether PACAP can counteract aging-induced changes. Indeed, several studies show that PACAP can act as an anti-aging peptide in several organs [55]. Most results come from PACAP-deficient mice, which show early aging signs. Ohtaki and coworkers found that PACAP-deficient mice have increased oxidative stress level with aging [90], while another study revealed several degenerative changes in the retinas of mice lacking endogenous PACAP [91]. In a recent study, presenile systemic amyloidosis was described in PACAP-deficient mice, including changes in the kidneys [56]. Systemic amyloidosis is characterized by deposition of amyloid fibrils in different tissues, finally leading to multiple organ dysfunction. PACAP-deficient mice showed severe amyloidosis in several organs, among them, one of the most affected organs was the kidney, in addition to the spleen, intestines, thyroid gland, esophagus, skin and liver. In the kidneys, amyloid deposits were observed in the renal corpuscles from very early age: one-third of the animals between ages 3–12 months already showed signs of amyloidosis in the kidney, in contrast to wild type mice, where no amyloid deposits were observed at young age. At older ages, nearly 90% of the PACAP-deficient mice displayed severe amyloidosis in the kidney, while only half of the wild type mice showed this sign, in a less severe form. Altogether, presenile systemic amyloidosis was present in mice lacking endogenous PACAP, affecting more individual animals at younger ages, in a more generalized and more severe form than in wild-type mice. The renal failure was also reflected in increased serum creatinine levels in aging PACAP-deficient mice [56]. In summary, lack of endogenous PACAP sensitizes the kidney and several other organs for accelerated amyloid deposition.

## 4. Concluding Remarks

The present review summarized in vitro and in vivo data showing the nephroprotective effects of PACAP. Based on these data, PACAP is a promising therapeutic agent in both acute and chronic renal pathologies and can potentially alleviate nephrotoxicity induced by various drugs with nephrotoxic side effects and can also ameliorate damage in chronic nephropathies, like diabetic nephropathy. There is very little known whether these actions observed in vitro and in animal studies in vivo are also present in the human kidney. There are very few studies addressing the presence and functional role of PACAP in the human kidney. A recent study by Eneman et al. [92] found VPAC receptors in normal and nephrotic kidneys and described increased PACAP immunoreactivity in the proximal tubules of nephrotic children, pointing to the reabsorption of filtered PACAP. Another study found decreased PACAP immunoreactivity in tissue samples from renal cancer patients [93]. Whether the protective effects of PACAP proven in numerous models in vitro and in vivo can be of human use requires further investigation, but the data summarized in this review point to the potential of PACAP as an effective agent preventing and/or ameliorating kidney damages induced by several different stimuli.

## Figures and Tables

**Table 1:** Summary of the renoprotective effects of PACAP against different stressors. (↑ increasing effect, ↓ decreasing effect).

**Harmful Stimulus/Disease**	**Observed Effect**	**Reference(s)**
**In Vitro**		
oxidative stress	↓ oxidative stress-induced cell death in rat renal cells	[39]
cyclosporine A	↓ morphological changes ↓ cyclosporine a-induced TGF-β1 ↓ apoptosis	[40]
gentamicin	↓ gentamicin-induced cell death in HK-2 cells	[41]
cisplatin	↓ cisplatin-induced apoptosis in mouse renal proximal tubule cells	[42]
LPS exposure	↓ proinflammatory cytokines in podocytes	[43]
hypoxia	↓ cytokine activation ↓ cell death	[44]
kappa light chains	↓ IL-6, TNF-α ↓ p38 phosphorylation	[45]
**In Vivo**		
Ischemia/reperfusion	↑ renal function and kidney morphology in warm ischemia	[46]
	↓ tubular degeneration and mortality	[47]
	↓ I/R-induced increase of CNTF, fractalkine, sICAM-1, RANTES, TIMP-1 and MIP-3α ↓ Bcl-2	[48]
	↓ SOD and glutathione	[39]
	↓renal failure, histological damage, neutrophil influx and tubule cell apoptosis	[44]
	gender-different protective effect ↓ tubular damage ↓ fractalkine, L-selectin, RANTES, sICAM-1, thymus chemokine, CNTF ↑ SOD in females influences BMP signaling	[49]
	in PACAP deficiency after I/R: ↓ SOD level more severe histological damage elevated expression of: BLC, G-CSF, IL-1ra, IL-6, KC, MCP-1, MIP-2, TIMP-1, TREM-1 decreased expression of: sICAM-1, interferon-γ, IL-1α, IL-1β, IL-2, IL-3, IL-4, IL-7, IL-10, IL-13, IL-16, IL-17, IL-23, IL-27, IP-10, M-CSF, MCP-5, MIG, SDF-1	[50,51]
cyclosporine A	↓ apoptosis ↓ renal injury and fibrosis ↓ extracellular matrix mRNA expression reverse activation of epithelial mesenchymal transition markers ↓ ROS	[40]
diabetic nephropathy	↓ histological damage ↓ cytokine activation	[52,53]
	↓ TNF-α	[53]
	↑ pAkt, pERK1/2 ↓ collagen IV, TGF-β-1 ↓ pp38MAPK, cleaved caspase-3 ↓ p60 subunit of NFκB ↑ GSH no focal segmental glomerular basement membrane thickening ↓ podocyte injury	[54]
gentamicin	↓ TNF-α production	[53]
cisplatin	↓tubular damage ↑renal function ↑ tubular cell regeneration	[42]
amyloidosis	pre-senile amyloidosis in PACAP deficiency	[55,56]
